# Clinical application of auricular point sticking in perioperative hemostasis for elderly patients with intertrochanteric fractures of the femur: Erratum

**DOI:** 10.1097/MD.0000000000018593

**Published:** 2019-12-20

In the article, “Clinical application of auricular point sticking in perioperative hemostasis for elderly patients with intertrochanteric fractures of the femur”,^[1]^ which appears in Volume 98, Issue 35 of *Medicine*, Chunfang Yin and Jincun Zhang contributed equally to the article. Also, Zhaojuan Er's affiliation should be “Department of Rehabilitation, Tianjin Baodi Hospital, Baodi Clinical College of Tianjin Medical University, Tianjin 301800, China.”
